# An In Vitro Investigation into Cryoablation and Adjunctive Cryoablation/Chemotherapy Combination Therapy for the Treatment of Pancreatic Cancer Using the PANC-1 Cell Line

**DOI:** 10.3390/biomedicines10020450

**Published:** 2022-02-15

**Authors:** John M. Baust, Kimberly L. Santucci, Robert G. Van Buskirk, Isaac Raijman, William E. Fisher, John G. Baust, Kristi K. Snyder

**Affiliations:** 1 CPSI Biotech, Owego, NY 13827, USA; ksantucci@cpsibiotech.com (K.L.S.); rvanbus@binghamton.edu (R.G.V.B.); ksnyder@cpsibiotech.com (K.K.S.); 2 GI Cryo, Inc., Owego, NY 13827, USA; 3 Center for Translational Stem Cell and Tissue Engineering, Binghamton University, Binghamton, NY 13902, USA; jgbaust@binghamton.edu; 4 Department of Biological Sciences, Binghamton University, Binghamton, NY 13902, USA; 5 Department of Medicine-Gastroenterology, Baylor College of Medicine, Houston, TX 77030, USA; raijman@bcm.edu; 6 Department of Medicine, University of Texas Health Science Center, Houston, TX 77030, USA; 7 Department of Surgery, Baylor College of Medicine, Houston, TX 77030, USA; wfisher@bcm.edu; 8 Elkins Pancreas Center, Dan L Duncan Comprehensive Cancer Center, Houston, TX 77030, USA

**Keywords:** ablation, cryosurgery, freeze dose, pancreatic cancer, gemcitabine, oxaliplatin

## Abstract

As the incidence of pancreatic ductal adenocarcinoma (PDAC) continues to grow, so does the need for new strategies for treatment. One such area being evaluated is cryoablation. While promising, studies remain limited and questions surrounding basic dosing (minimal lethal temperature) coupled with technological issues associated with accessing PDAC tumors and tumor proximity to vasculature and bile ducts, among others, have limited the use of cryoablation. Additionally, as chemotherapy remains the first-line of attack for PDAC, there is limited information on the impact of combining freezing with chemotherapy. As such, this study investigated the in vitro response of a PDAC cell line to freezing, chemotherapy, and the combination of chemotherapy pre-treatment and freezing. PANC-1 cells and PANC-1 tumor models were exposed to cryoablation (freezing insult) and compared to non-frozen controls. Additionally, PANC-1 cells were exposed to varying sub-clinical doses of gemcitabine or oxaliplatin alone and in combination with freezing. The results show that freezing to −10 °C did not affect viability, whereas −15 °C and −20 °C resulted in a reduction in 1 day post-freeze viability to 85% and 20%, respectively, though both recovered to controls by day 7. A complete cell loss was found following a single freeze below −25 °C. The combination of 100 nM gemcitabine (1.1 mg/m^2^) pre-treatment and a single freeze at −15 °C resulted in near-complete cell death (<5% survival) over the 7-day assessment interval. The combination of 8.8 µM oxaliplatin (130 mg/m^2^) pre-treatment and a single −15 °C freeze resulted in a similar trend of increased PANC-1 cell death. In summary, these in vitro results suggest that freezing alone to temperatures in the range of −25 °C results in a high degree of PDAC destruction. Further, the data support a potential combinatorial chemo/cryo-therapeutic strategy for the treatment of PDAC. These results suggest that a reduction in chemotherapeutic dose may be possible when offered in combination with freezing for the treatment of PDAC.

## 1. Introduction

An estimated 60,430 new cases of pancreatic cancer (PaCa) will be diagnosed in 2021 in the US alone [1]. With only a 10.8% 5-year survival rate for all stages combined, improved methods of detection and treatment are needed [1]. Signs and symptoms of weight loss, jaundice, and pain often only occur once late-stage cancer has developed, which makes early detection, and thus successful treatment, difficult. PaCa staging is complex and tumors are often categorized as resectable, borderline resectable, or unresectable, which includes locally advanced or metastatic disease, for treatment [2]. Resectable cancers are typically stage 1, limited to the pancreas only and divided into stage 1A (2 cm or smaller) and stage 1B (between 2 and 4 cm). Stage 2A may also be resectable (>4 cm) while stage 2B describes a cancer that has spread to the lymph nodes; increasing stage reflects a poorer prognosis. At the time of diagnosis, approximately 52% of patients present with distant metastasis, 30% with regional disease, and only 11% with disease localized to the pancreas [3]. Complete surgical removal is the only curative approach, but only a small number of patients fall into this category. Currently, treatment depends on staging, with resectable tumors typically surgically excised after the patient is treated for 6 months with neoadjuvant chemotherapy. In cases where surgical resection is not possible or is high risk due to tumor size, proximity to blood vessels, or other factors (non-resectable tumors), chemotherapy is the typical treatment, and sometimes radiation, small molecules, or immunotherapy are added. Thermal ablation techniques, such as radiofrequency (RFA) or cryoablation (CA), are sometimes utilized, though to a much lesser degree.

While several treatment options exist for non-resectable pancreatic ductal adenocarcinoma (PDAC), they are all considered palliative. Palliative treatment options for non-resectable PDAC include chemotherapy (gemcitabine, oxaliplatin, cisplatin, 5-FU, and taxanes), with the standard treatment for locally advanced PDAC being chemotherapy (FOLFIRINOX or Gem/Abraxane) with or without radiation [2], despite the significant complications [4]. Additionally, a number of small molecule kinase inhibitors (including Erlotnib, Sunitinib, and others) have also been approved for treating PaCa [5,6,7,8]. Thermal ablation, including radiofrequency ablation (RFA), microwave, high-intensity focused ultrasound (HIFU), and cryoablation (CA), are now being explored as treatment options for non-resectable PaCa [2]. Hyperthermal ablation (RFA, HIFU, and microwave) heats tissue to lethal temperatures (70 to 90 °C) and kills primarily by heat-induced damage such as membrane alterations, proteins denaturation, and necrosis, whereas cryoablation freezes tissue and kills through freeze rupture, necrosis, and apoptosis [9,10]. Recent technological advances and promising investigational studies support the use of cryoablation as a robust ablative therapy with demonstrated efficacy in a number of cancers, including prostate, kidney, liver, and breast, as well as metastatic lesions [11,12,13,14,15]. Cryoablation has also been extensively utilized for other nonresectable primary and secondary (e.g., colon, gastric) cancers [16,17,18,19].

Cryoablation is typically performed percutaneously, whereby one or more cryoprobes are inserted and the target tissue is frozen. The freezing process is completely destructive to the tissue closest to the cryoprobe where temperatures are coldest [20,21]. As the distance from the cryoprobe increases, a thermal gradient is generated with the coldest temperatures within the core and the edge of the frozen tissue being 0 °C, nominally. As a result, responses and stresses across the treatment zone differ. Cells close to the cryoprobe experience rapid cooling rates, ultralow temperatures (<−40 °C), and an increased probability of intracellular ice. Cells more distant from the probe (>−40 to <−10 °C) primarily experience extracellular ice, whereas cells even more distal experience only hypothermia (>−10 °C). This results in the activation of multiple modes of cell death (ice rupture, necrosis, and apoptosis) within a cryogenic lesion [22,23,24]. Further, the resultant warmer temperatures in the periphery of the cryogenic lesion are often insufficient to yield complete cell death and have the potential to result in tumor recurrence [25]. As previously reported, the lethality of a cryolesion is dependent on several factors, including the cryogen (nitrogen, argon, nitrous oxide, etc.) and the resultant tip temperature, surface contact, cooling rate, length of freeze, number of freeze–thaw cycles, proximity to major vasculature, tumor size, and, importantly, cell sensitivity to freezing [26,27].

Cryoablation has primarily been used as palliative care in metastatic PaCa, but a small number of studies have found encouraging results, especially when combined with adjunctive techniques, including immunotherapy [28,29]. Cryoablation achieves the goal of debulking tumor mass while being less invasive than traditional surgery. A percutaneous approach using a cryoprobe can produce multiple cryolesions in a single procedure. Due to the location of the pancreas, targeting many PaCa tumors for percutaneous-based thermal ablation can be difficult. Recently, the development of endoscopic ultrasound (EUS)-guided thermal ablation devices and techniques are now enabling a less invasive treatment path, a significant advantage over traditional surgical methods, and potentially allowing for greater patient inclusion. A number of small studies have been conducted to evaluate the feasibility of EUS-guided thermal ablative therapies for pancreatic targets. Pai et al. [30] published the first human pilot study using an EUS-compatible RFA probe to treat PNET and concluded the procedure was straightforward and safe. Since then, several EUS-compatible probes have been developed which can deliver RFA, microwave, or laser ablation to pancreatic targets [31]. More recently, our group has developed a next-generation cryoablation system and EUS-compatible cryocatheter, further expanding the potential for ablative therapies in PaCa [32].

Freezing has been shown to be as, or more, effective and safer than heat-based ablation in several indications [33,34,35,36]. In a study by Chiu et al. [37] on PaCa cryoablation, no edema or hemorrhage was observed in the non-frozen areas, whereas histological assessment of the frozen region revealed significant necrosis with all cellular ultrastructures destroyed, prompting the group to declare that cryoablation is a safe and effective ablative procedure for pancreatic tissue. Similar positive outcomes have been reported by Petrone et al. [38] and Kovach et al. [38]. These, and other, reports demonstrate the potential for cryoablation to treat PaCa [28,39,40]. Given that some PaCa tumors are poor candidates for surgical resection due to proximity to blood vessels and other organs, the precise targeting of cancerous cells with cryoablation could expand therapeutic options to more patients.

With cryoablation emerging as a treatment option for PaCa as well as the continued desire for improved therapeutic options, interest in the combination of cryoablation with other treatment modalities, such as anti-cancer agents, is growing. Studies by Xu et al. [41,42] have reported positive outcomes of the combination strategy of cryoablation and radioactive seeds for PaCa. A recently completed clinical trial (NCT03695835) on the combination of cryoablation and pembrolizumab included a PaCa subgroup with initial results showing a benefit. We, as well as others, have published numerous studies on the effects of the combination of freezing with adjunctive agents, including chemotherapeutic drugs and nutraceuticals, in various cancer cell systems [40,41,42,43,44,45,46,47,48,49,50,51,52,53,54]. The objective of this combinatorial adjunctive agent approach is to elevate the minimum lethal temperature necessary to fully ablate a tumor, thus ensuring complete cancer cell destruction with minimal over-freezing. To date, studies have focused on prostate, renal, lung, and liver cancers, characterizing the impact of combination treatment on cell survival. Importantly, these studies have demonstrated that the impact of combination treatment varies significantly based on the cancerous tissue type, molecular variant, drug utilized, etc. [22,23,25,32,55]. Further, investigation into the molecular pathways responsible for the reported benefits of combination remain in their infancy. Lastly, no studies characterizing the response of PaCa to cryotherapy or the impact of combinatorial chemotherapy/cryoablation on PaCa have been reported.

Given the promise for cryoablation to treat PaCa combined with a growing interest in adjunctive treatment strategies, in this study we investigated the freeze response of PaCa in vitro using the PANC-1 cell line in an effort to identify the lethal temperature as well as investigate the effect of the combination of cryoablation and chemotherapy for improving cancer destruction. PANC-1 cells are a well-accepted cell model for in vitro PaCa studies. PANC-1 cells are pleomorphic and are reported to express CK5.6, MNF-116, vimentin, chromogranin A, CD56, and SSTR2, but not E-cadherin, synaptophysin, or NTR1. PANC-1 cells are CD24−/+, CD44+, CD326−/+, and CD133/1− and have KRAS and TP53 mutations and homozygous deletions, including the first three exons of CDKN2A/p16INK4A, but no SMAD4/DPC4 mutations [56,57]. Studies have shown PANC-1 cells to be highly invasive (metastatic) and tumorigenic [57,58,59]. PANC-1 cells were selected for the current study as previous reports have shown PANC-1 cells to be much more resistant to ablation (both freezing and heating) compared to other PaCa cells such as BxPC3 [60,61,62]. Further, studies have shown PANC-1 cells to be more resistant to chemotherapy, including gemcitabine, compared to other PaCa cell lines including BxPC3 and MIA-PANC-2 cells [59,63]. Given the reported increased resistance to ablation and chemotherapy, we utilized PANC-1 cells in an effort to develop a new treatment strategy for this difficult-to treat PaCa variant. The use of adjuvants in combination with freezing to sensitize cells in the ice ball periphery prior to or during a freezing event is an area of growing interest [41,42,45,47,48,51,52,53,54,64,65,66,67,68,69,70,71,72]. There are a number of cytotoxic agents used in the treatment of PaCa, including gemcitabine and/or oxaliplatin. These agents are typically administered systemically at doses of 800–1000 mg/m^2^ and up to 130 mg/m^2^, respectively [73,74,75]. Gemcitabine was once a first-line treatment; however, many patients developed chemoresistance quickly when it was used as a monotherapy. As such, the use of drug combinations (FOLFIRINOX or Gem/Abraxane) are now typical first-line treatments. Additionally, the use of small molecule kinase inhibitors to treat chemoresistant PaCa has been reported to yield benefit [7,8]. Regardless of the agent(s), at clinical doses, the well-known side effects of chemotherapy can adversely affect patient quality of life. The combination of cryoablation with either systemic or infusion-based chemotherapy may allow for reduced levels of the drug needed to achieve complete cancer destruction within a frozen mass, thus enabling the possibility of a curative response. In this study, we also investigated the impact of the pre-treatment of PANC-1 cells with subclinical doses of gemcitabine or oxaliplatin followed by freezing. Lastly, we investigated the role of apoptotic cell death (caspase-3 activation) in PaCa cell death following freezing, low-dose gemcitabine exposure, and combination treatment. We hypothesized that the application of a subclinical (non-lethal) dose of a chemotherapeutic agent prior to freezing would act synergistically, thereby increasing cell destruction, via increased apoptotic caspase activity, at typically non-lethal temperatures while reducing the overall negative effects associated with typical clinical chemotherapy doses.

## 2. Materials and Methods

### 2.1. Cell Culture

PANC-1 cells (a human pancreatic cancer cell line isolated from a pancreatic carcinoma of ductal cell origin, ATCC- CRL 1469 (Manassas, VA, USA)) were cultured in DMEM (Caisson DML10 (Smithfield, UT, USA)) with 10% FBS (Peak Serum, Wellington, CO, USA)) and 1% penicillin/streptomycin (Lonza, Walkersville, MD, USA). Cultures were maintained in T-75 flasks in 95% air/5% CO_2_ incubators and passaged at 80% confluence. Cells were plated into costar strip well plates at 15,000 cells per well and cultured for 3 days prior to freezing.

### 2.2. Tissue-Engineered Model Generation

Rat tail type I collagen solution (BD Bioscience, Bedford, MA) was used to form 0.2% *w*/*v* gel matrices as per SOP. Cells (0.75–1 × 10^6^ cells/mL) were suspended in the liquid collagen solution within 3 mm × 40 mm tissue-engineered model (TEM) ring fixtures, then placed in 100 mm petri dishes and allowed to solidify for 30 min in a 37 °C hybridization oven as per Robilotto et al. and Baust et al. [27,76,77,78]. Following gelation, 15 mL of cell culture medium was added to the dish to cover the TEMs and the dishes were placed in the incubator. The TEM cells containing matrices were cultured for 24 h prior to 48-h drug exposure where indicated, or 72 h total, prior to utilization.

### 2.3. Drug Treatment

Gemcitabine (Sigma-Aldrich #G-6423, St. Louis, MO, USA) was prepared fresh in sterile water prior to each use and diluted to final sub clinical concentrations in media. The concentrations chosen for the dose response study were 10, 50, 100, 500, 1000, and 5000 nM gemcitabine for 48 h. The clinical equivalencies were 0.11, 0.56, 1.11, 5.57, 11.1, and 55.5 mg/m^2^, respectively. In vivo doses typically range from 800 to 1000 mg/m^2^ in a single intravenous weekly treatment, with treatment cycles often occurring weekly for 8 weeks. Gemcitabine doses of 10, 50, and 100 nM were selected for freeze response combination studies. The drug was applied as described above for 48 h and removed 30 min prior to freezing.

Oxaliplatin (Sigma-Aldrich #O-9512, St. Louis, MO, USA) was prepared as a 5 mM stock solution in sterile water and stored at −20 °C. Stock solutions were thawed and diluted to final concentrations in media. Dose response study concentrations were 0.88, 1.5, 3, 5, 8.8, and 10 µM oxaliplatin with a 48-h exposure. The clinical equivalencies were 13, 22, 45, 73, 130, and 150 mg/m^2^, respectively, with a typical in vivo clinical dose of 130 mg/m^2^. Oxaliplatin doses of 3, 5, and 8.8 µM were selected for freeze response combination studies. The drug was applied as described above for 48 h and removed 30 min prior to freezing.

### 2.4. Freezing Protocol

#### 2.4.1. Cell Culture Freeze Procedure

Samples in Costar 8-well strips (75 µL medium/well) were exposed to freezing temperatures of −15 °C in a refrigerated circulating bath (Neslab/Thermo Scientific, Waltham, MA, USA) for 5 min. Thirty minutes prior to freezing, culture medium was aspirated and replaced with 75 µL per well of appropriate culture medium. Strips were placed into aluminum blocks, containing a thin coating of ethanol to facilitate complete contact and thermal exchange with each well, within the baths. Sample temperature was monitored in a cell-free well and ice nucleation was initiated at −2 °C using liquid nitrogen vapor to prevent supercooling. Sample temperature was recorded at 1-s intervals using a type T thermocouple (Omega HH806AU, Omega, Stamford, CT, USA). For freezing, samples were held for a total time of 5 min in the freezing bath, passively thawed at room temperature for 10 min under a laminar flow hood, and then placed at 37 °C for recovery and assessment. 

#### 2.4.2. TEM Freeze Procedure 

All tests were performed in a laminar flow hood to ensure sample sterility. Freezing was conducted using the PSN (Pressurized Sub-Cooled Nitrogen) Cryosystem (CPSI Biotech, Owego, NY, USA) with an input N_2_ pressure of 1500 psi [79]. The cryoablation probe utilized in these studies was a 1.5 mm diameter cryoprobe with a 3 cm long freeze zone at the distal end (FrostBite, CPSI Biotech). 

Individual cell-seeded TEMs were stacked into the 3-D configuration as detailed in Robilotto et al. [80] and submerged in warm circulating culture medium within an acrylic box. The box was placed onto a heat pad and stir table and the cryoprobe and thermocouple array consisting of four type T thermocouples were inserted into the fixture. Samples were held until TEM and bath temperatures equilibrated at 32 °C (±2 °C). TEMs were frozen using a single 5-min freeze protocol. The temperature of the bath and within the TEM was monitored throughout the freezing process at the midpoint of the freeze zone using a type-T multipoint thermocouple array at fixed distances of 7.5 mm, 10.5 mm, 13 mm, and 16 mm extending radially from the surface of the cryoprobe. Temperatures were recorded using an Omega TempScan at 10 s intervals throughout the entire freeze cycle. At the completion of the freeze cycle, TEMs were allowed to passively thaw in the warm circulating bath for 30 min prior to disassembly, at which time the individual TEM layers were returned to culture for recovery and assessment.

### 2.5. Cell Viability and Data Analysis

#### 2.5.1. Two-Dimensional Viability Assessment and Analysis

The metabolic activity indicator alamarBlue (Invitrogen, Carlsbad CA, USA) was utilized to assess cell viability. Stock alamarBlue was diluted 1:20 in Hank’s Balanced Salt Solution (HBSS, Corning/Mediatech (Corning, NY, USA)) and applied to samples for 60 min (±1 min) at 37 °C. Raw fluorescent units were obtained using a TECAN Infinite plate reader (excitation 530 nm and emission of 590 nm, Tecan Austria GmBH, Grodig, Austria) and analyzed using Microsoft Excel. Raw fluorescence units were converted to percentages based upon pre-freeze control values (±SEM). Assessments were conducted on day 1, 3, 5, and 7 of recovery. 

A minimum of 3 experimental repeats with an intra experimental repeat of 7 wells was performed in each condition (n ≥ 21). Statistical significance was determined by single-factor ANOVA where *p* < 0.01 was applied as the significance threshold.

#### 2.5.2. TEM Viability Assessment and Analysis

Following thawing, individual TEM layers were measured via calipers to determine the diameter of the frozen tissue (ice ball) created following the freeze–thaw cycle. Ice ball radii were measured at cardinal locations around the probe surface to determine the symmetry of the freeze zone created. TEMs were then placed into culture to assess at 24 h post-freezing recovery. In situ sample viability assessment (live/dead assay) was performed to determine the extent of cell death (cryolesion) using the fluorescent probes calcein-AM and propidium iodide (Cal/PI; Molecular Probes, Eugene, OR). Briefly, culture medium was decanted from the TEM samples and a working solution of 5 µg/mL calcein-AM (live cells; Molecular Probes) and 4 µg/mL propidium iodide (necrotic cells; Molecular Probes) in 1× PBS (Corning) was added directly to each sample. Samples were incubated in the dark at 37 °C for 60 min (±1 min). Fluorescent staining was visualized using a Zeiss Axio Observer 7 with ZEN software (Carl Zeiss AG, Oberkochen, Germany). Panoramic digital images spanning the center of the freeze zone were acquired using a 1× objective and stitched together from a 6 × 30 set of overlapping images. Following acquisition, a scale bar was imprinted onto each of the images to enable direct image comparison. Diameters of the necrotic zones were then measured using the ZEN software measurement tool. 

All experiments were repeated a minimum of 3 times. Following testing, data were combined and averaged (±standard deviation) to determine mean ice ball size, isotherm distribution, and ablative diameter. Statistical significance was determined using single-factor ANOVA where noted.

### 2.6. Immunofluorescent Analysis of Cleaved Caspase-3 Activity

Samples were fixed in situ with methanol at day 1, 3, and 5 post-freeze. Briefly, media was aspirated and samples were rinsed with 1x PBS. Ice cold methanol was applied dropwise and samples were placed at −20 °C for 15 min. Methanol was removed and samples were rinsed three times with 1× PBS for 5 min each. Samples were exposed to blocking buffer (5% normal goat serum with 0.3% triton-X-100 in 1× PBS) for 60 min at room temperature followed by incubation in primary antibody (cleaved caspase-3, Novus NB 500-1235, 1:200 dilution(Novus, Centennial, CO, USA)) at 4 °C overnight. Secondary antibody (Alexa Fluor 488 conjugated goat anti-rabbit secondary antibody at 1:500 dilution (Cell Signaling Technology, Danvers, MA, USA)) was applied for 60 min at room temperature and samples were rinsed three times with 1× PBS for 5 min each. Samples were counterstained with Hoechst 33342 (Invitrogen, 10 mg/mL) for 1 min prior to image acquisition. Samples were covered with 1× PBS and scanned using a CX5 CellInsight High Content Screening device (Thermo Fisher Scientific) with HCS studio software. Resultant images (10 × 10 mosaic of each individual well) were quantified using the Compartmental Analysis bioapplication with all reference levels set based on control samples, which allowed for identification of the subcellular location of active caspase-3. Immunofluorescent experiments were repeated a minimum of 3 times with an interexperimental analysis of a minimum of 3 wells/condition/experimental repeat. Data from 9 individual samples were combined and then converted to percentage (±SD) of cells positive for the nuclear localization of cleaved caspase-3 based on the total cell population present in condition.

## 3. Results

### 3.1. Pancreatic Cancer Cell Response to Cryoablation

Acellular hydrogels are often utilized to assess the generation and spread of critical isotherms and the cooling power of cryoprobes. While useful, these models do not provide information on the response of cells or tissues to the freezing regime. To bridge this gap, we have previously reported on the use of in vitro, three-dimensional tissue constructs (TEM) as a clinically analogous test setup to provide for evaluation of the thermal performance of a cryoprobe as well as the assessment of the level of cell death associated with a given protocol [27,76,77,78,80,81,82]. This model has been shown to provide vital information on in vivo response while reducing the expense and burden of exploratory animal studies [27,79,80]. 

**Figure 1:**
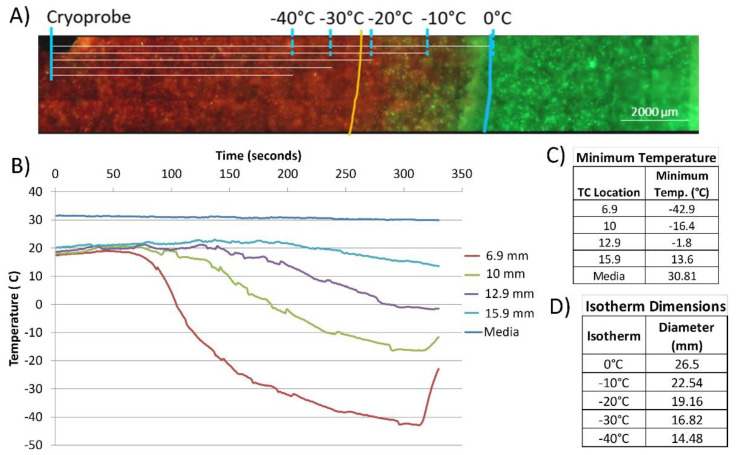
Assessment of cell death following cryoablation using a PaCa TEM. (**A**) Fluorescent micrograph of the PANC-1 TEMs following single 5 min freeze. At 24 h post-freeze, TEMs were probed with calcein-AM (green, live) and propidium iodide (red, dead) and visualized using fluorescent microscopy to determine the extent of cell death. Isotherms were extrapolated from thermal monitoring and imprinted onto images for assessment. The blue line represents the edge of the iceball and the orange line represents the edge of necrotic cell death. (**B**) Real-time monitoring of the isothermal profile generated by the PSN 1.5 mm cryoprobe at the center of the ablation segment within a TEM during a 5-min freezing protocol. Temperatures of the bath (“media”) and within the TEM were monitored throughout the freezing process at the midpoint of the freeze zone using a type-T multipoint thermocouple array at fixed distances extending radially from the surface of the cryoprobe. (**C**) Actual distances were measured using Zen software from images acquired from the visualized thermocouples within each TEM layer. (**D**) Isotherms (0 °C, −10 °C, −20 °C −30 °C, −40 °C) were extrapolated from measured data points.

TEMs consisting of PANC-1 cells were prepared and then treated with a single freeze. TEMs were frozen using a 1.5 mm x 3 cm PSN cryoneedle (FrostBite) under a single 5-min freeze protocol in a circulating heat loaded model. The thermal profile within the TEM during the freeze procedure was measured in real-time at fixed positions radiating from the center of the cryoprobe freeze zone (Figure 1a). TEMs were stained with calcein-AM (green, live) and propidium iodide (red, necrotic) at 24 h and 72 h post-thaw to determine the extent of cell death within the frozen volume (cryolesion) (Figure 1b). As calcein-AM and propidium iodide are single end-point assessments, sister samples were used to assess viability following 24 h of recovery. Representative fluorescent images in Figure 1 illustrate necrotic (red) versus live (green) regions within the TEMs following treatment. Isotherms from the thermal monitoring are imprinted over the image and the orange line represents the transition from variable survival to complete necrosis. Non-frozen control TEMs revealed no cell death (Figure 1b). Measurements of the ice ball diameter and cryolesion (cell death) in frozen/thawed TEMs were made using the ZEN software and results were averaged from triplicate experiments (Table 1). Measurements were obtained at 24 h and converted to volume of an ellipsoid using the length of the cryoprobe freeze zone. The percentage of the frozen mass that was completely ablated was then calculated (Table 1). 

Following a single 5 min freeze, the frozen diameter was 2.56 cm (±0.12 cm) equating to a volume of 13.55 cm^3^ (±0.3). The average size of the frozen areas measured on the micrographs corresponded to within 1 mm of those obtained with the thermal profile data collected during the freeze procedure. Following 24 h of recovery, TEMs had a zone of necrosis of 2.01 cm (±0.18) in diameter equating to a volume of 7.1 cm^3^ (±0.2 cm) after a single freeze. The area of complete destruction was found to be between the −20 °C and −25 °C isotherms and, when compared to the overall frozen volume, equated to −52.4% of the frozen mass being destroyed (necrotic). After 72 h of recovery, the necrotic diameter was found to be similar to 24 h post-freeze with a diameter of 1.99 cm (±0.18 cm) equating to 7.01 cm^3^ (±0.25 cm) or 53.2%. 

**Figure 2:**
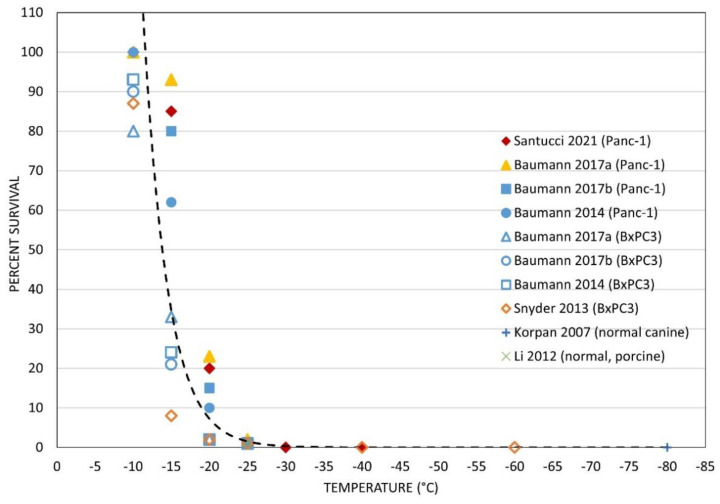
Comparison of the reported level of PaCa cell death following freezing. Data on pancreatic cell death following exposure to various freezing temperatures were collected from the literature and overlaid with specific survival data found following PANC-1 TEM freezing. The TEM data correlated well with previous in vitro and in vivo findings. Trend analysis revealed complete cell death below −25 °C and a transitional level of cell death between −15 °C and −25 °C.

With the identification of complete ablation below −25 °C and a zone of partial ablation between −20 °C and −10 °C in the TEM model, we compared our findings to those previously reported in the literature in various pancreatic models [60,61,62,83,84,85]. Plotting the reported survival from the pancreas cryoablation literature revealed that the TEM results correlated well (Figure 2). Specifically, previous in vitro studies by Bauman et al. [60,61] (blue symbols) as well as Snyder et al. [62] (orange symbols) using the PANC-1 (solid symbols) and BxPC3 (open symbols) PaCa cell models reported a high level of cell survival following freezing to temperatures of ≥−15 °C, substantial yet not complete cell death following freezing to −20 °C (<30% survival), and near-complete cell death (<2% survival) following freezing to −25 °C, whereas at <−30 °C complete cell death was found (Figure 2). Near-complete pancreatic cell death following freezing to −25 °C has also been reported by Li et al. [85] (x symbol). These reports were similar to our TEM findings (solid diamond), wherein ~85% survival was estimated at the −15 °C isotherm, −20% at −20 °C, and complete cell destruction below −30 °C. 

### 3.2. Drug Exposure Dose Response of Pancreatic Cancer Cells In Vitro

#### 3.2.1. Dose Response of PANC-1 Cells to Gemcitabine In Vitro

**Figure 3:**
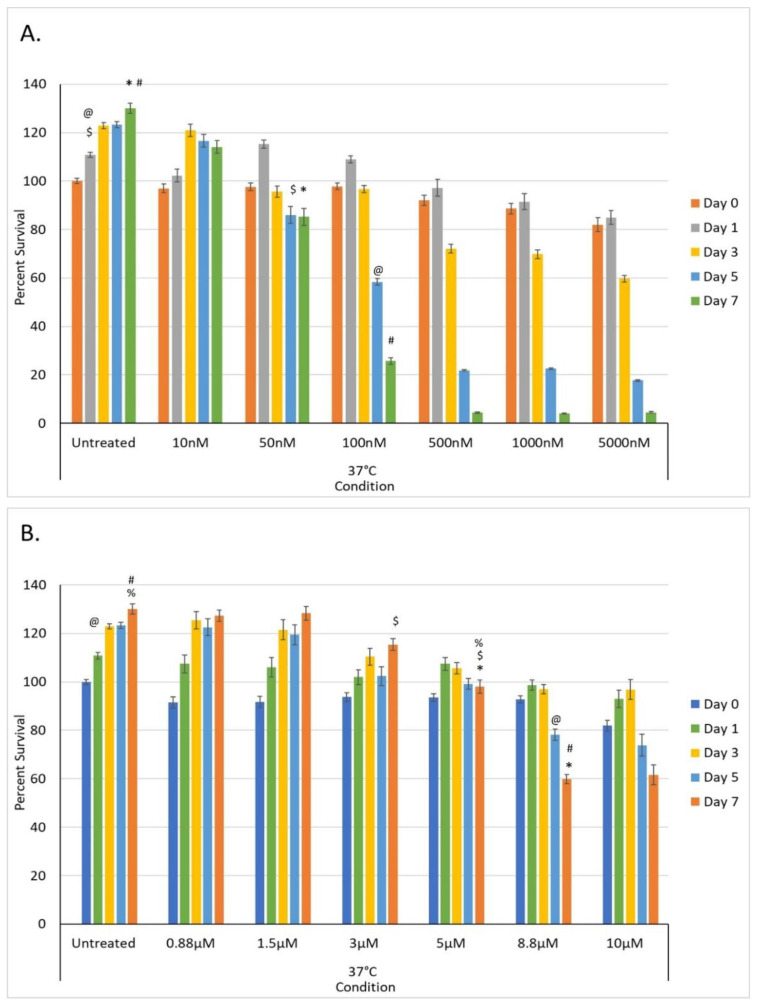
Assessment of the impact of low-dose chemotherapeutic drug exposure on PaCa cell viability in vitro. (**A**) PANC-1 samples were exposed to 10, 50, 100, 500, 1000, and 5000 nM gemcitabine for 48 h. Data revealed that exposure to concentrations <50 nM had minimal impact on cell survival whereas >100 nM resulted in a gradual decline in viability over the 7-day assessment interval. (**B**) PANC-1 samples were exposed to 0.88, 1.5, 3, 5, 8.8, and 10 µM oxaliplatin for 48 h. Data revealed that exposure to concentrations <5 µM had minimal impact on cell survival whereas >8.8 µM resulted in a gradual decline in viability over the 7-day assessment interval (@, #, %, $, * = *p* < 0.01).

A dose response study was conducted to determine the impact of a 48-h exposure of PANC-1 cells to gemcitabine at concentrations of 10, 50, 100, 500, 1000, and 5000 nM (Figure 3). The concentrations evaluated equate to clinical doses of 0.11, 0.56, 1.11, 5.57, 11.1, and 55.5 mg/m^2^, respectively. A typical clinical dose for gemcitabine for PaCa is 800–1000 mg/m^2^; as such, the evaluated concentrations represented a significantly lower dose than typically utilized clinically. 

Assessment of PANC-1 samples following 48 h exposure to 10, 50, 100, 500, 1000, and 5000 nM gemcitabine revealed sample viabilities of 97.0% (±1.8), 97.6% (±1.7), 97.8% (±1.4), 88.6% (±2.2), and 82.1% (±2.8) of untreated control samples (100% (±1.0)), respectively, at the time of drug removal (day 0) (Figure 3a). Assessment over the 7-day recovery period revealed that sample viability declined following exposure to doses of ≥100 nM by day 7, whereas treatment with 10 nM and 50 nM gemcitabine resulted in minimal to no cell death by day 7. Specifically, day 7 sample viability measured 114.1% (±2.1), 85.2% (±3.5), 25.7% (±1.3), 4.4% (±0.3), 4.1% (±0.3), and 4.5% (±0.3), respectively, versus non-treated controls (130.1% (±2.1)).

#### 3.2.2. Dose Response of PANC-1 Cells to Oxaliplatin In Vitro

**Figure 4:**
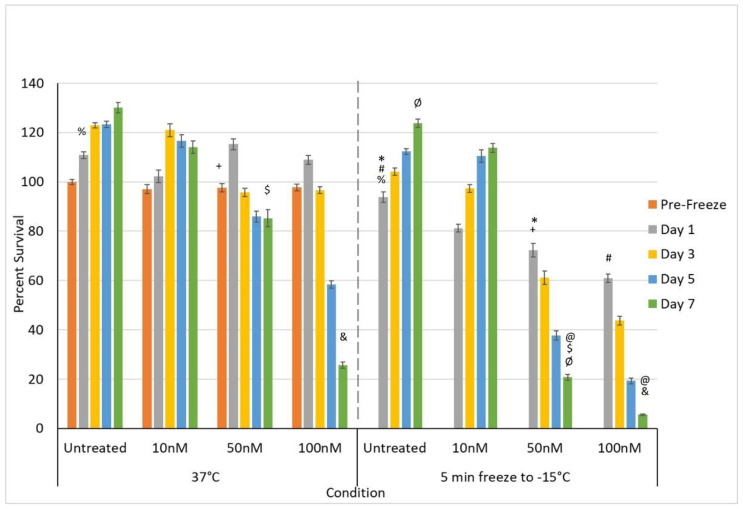
Effect of adjunctive gemcitabine pretreatment at sub-clinical dose in combination with freezing on PaCa cell survival. Samples were pre-treated with 10, 50, or 100 nM gemcitabine for 48 h and drug removed prior to freezing to −15 °C. Sample viability was compared to freezing and gemcitabine treatment alone. Data reveal a significant improvement in cell death following the combination of 50 and 100 nM gemcitabine and −15 °C, whereas 10 nM gemcitabine and −15 °C samples showed no benefit. Importantly, the combination of 100 nM gemcitabine and −15 °C yielded complete cell death by day 7 (@, #, $, %, &, *, +, Ø = *p* < 0.01).

A dose response study was also conducted to determine the impact of a 48-h exposure of PANC-1 cells to oxaliplatin at concentrations of 0.88, 1.5, 3, 5, 8.8, and 10 µM (Figure 4). The concentrations evaluated equate to clinical doses of 13, 22, 45, 73, 130, and 150 mg/m^2^, respectively. A typical clinical dose for oxaliplatin for PaCa is 130 mg/m^2^; as such, several of the evaluated concentrations were significantly lower than a typical clinical dose. 

Assessment of PANC-1 samples following 48 h exposure to revealed sample viabilities of 91.5% (±2.3), 91.7% (±2.3), 93.7% (±1.8), 93.5% (±1.6), 92.7% (±1.5), and 81.9% (±2.2) of untreated control samples (100% (±1.1)) at the time of drug removal (day 0) (Figure 3b). Assessment over the 7-day recovery period revealed that sample viability declined in the 8.8 µM and 10 µM conditions over the 7-day assessment period, whereas 0.88, 1.5, 3, and 5 µM resulted in minimal to no cell death. By day 7, sample viability measured 127.4% (±2.4), 128.4% (±2.8), 115.4% (±2.4), 98.1% (±2.8), 59.9% (±2.0), and 61.6% (±4.1), respectively, versus matched non-treated control samples (130.1% (±2.1)).

### 3.3. Impact of Adjunctive Chemo Pretreatment and Freezing on Pancreatic Cancer Cells

Following the confirmation of a significant level of cell survival following freezing to −15 °C and a precipitous drop in survival following freezing to −20 °C in the TEM model coupled with dose response studies showing a mild effect, a series of investigations into the impact of the combination of pre-treatment of PANC-1 cells with chemotherapeutic agents followed by freezing to −15 °C were conducted. These studies were conducted with the goal of obtaining complete PANC-1 cell death following freezing at −15 °C. Previous reports by our group as well as others have shown that this cryoablation/chemotherapy combination approach can significantly increase cell death following freezing to warmer sub-lethal temperatures [41,42,45,47,48,51,52,53,54,64,65,66,67,68,69,70,71,72]. Further, these reports have demonstrated that through the adjunctive cryoablation/chemotherapy combination approach, complete cancer cell death can be obtained following pretreatment with subclinical (ineffective) chemotherapy doses [41,48,52,64,86]. As such, we investigated the use of gemcitabine and oxaliplatin in combination with freezing to −15 °C.

#### 3.3.1. Impact of Adjunctive Gemcitabine Pretreatment and Mild Freezing 

Given the observed minimal decrease in PANC-1 viability following exposure to 10 and 50 nM gemcitabine (Day 7 survival >80%) as well as a significant level of PANC-1 survival following treatment with 100 nM (~25%), we explored the impact of combining gemcitabine pretreatment at these concentrations followed by freezing to −15 °C. The 10, 50, and 100 nM gemcitabine conditions were selected in an effort to evaluate the potential of reducing the drug concentration, thereby reducing the negative side effects experienced clinically while still yielding enhanced cell death when combined with freezing. Further, these doses were selected as none of them were completely lethal (e.g., 100% cell death) and they covered a range of cell death from none (10 nM) to mild (50 nM = ~20% death) to extensive (100 nM = ~75% death) as determined in the dose response studies (Figure 3a). Similarly, freezing to −15 °C was selected as the literature and TEM data revealed a mild level of cell death (<30%) (Figure 1 and Figure 2). 

Accordingly, samples were pretreated with 10, 50, or 100 nM gemcitabine for 48 h and then frozen with a single 5-min freeze to −15 °C (Figure 4). PANC-1 cells exposed to a single 5-min freeze at −15 °C revealed a minimal cell death with viability at day 1 post-freeze of 93.9% (±1.4) compared to controls (*p* < 0.01(%)). The surviving population rapidly recovered, reaching non-treated control levels by day 3.

Given the observed high level of PANC-1 viability and subsequent recovery following exposure to −15 °C as well as varying responses to 10, 50, and 100 nM gemcitabine, we explored the impact of combining gemcitabine pretreatment at these concentrations followed by freezing on cell survival (Figure 4). The combination of 10 nM gemcitabine and freezing yielded a slight increase in PANC-1 cell death compared to either treatment alone, yet the remaining population was able to recover and return to control levels by day 3 (Figure 4). When samples were treated with the combination of 50 nM gemcitabine pretreatment and freezing to −15 °C, a significant decrease in cell viability was noted compared to either condition alone as well as to the 10 nM combination samples. Specifically, PANC-1 cells exposed to 50 nM gemcitabine for 48 h followed by a single freeze at −15 °C resulted in a decrease in cell survival to 72.3% (±2.7), which was significant when compared to −15 °C freeze alone (93.0% (±1.4); *p* < 0.01(*)), 50 nM gemcitabine alone (97.6% (±1.7); *p* < 0.01(+)) or the −15 °C/10 nM gemcitabine combination condition (81.2% (±1.6); *p* < 0.01). Importantly, assessment of PANC-1 survival over the 7-day recovery period revealed a continued decline in the −15 °C/50 nM gemcitabine combination samples which differed from either condition alone. Specifically, PANC-1 cells exposed to 50 nM gemcitabine/−15 °C freeze combination resulted in a continued decrease in cell survival to 20.7% (±1.1), which was significant when compared to the day 7 viability of −15 °C freeze alone (123.7% (±1.6); *p* < 0.01(Ø)), 50 nM gemcitabine alone (85.2% (±3.5); *p* < 0.01($)), or the −15 °C/10 nM gemcitabine combination conditions (113.8% (±1.8); *p* < 0.01). This represented a ~3-fold increase in cell death versus 50 nM gemcitabine alone and a 4.5-fold increase compared to the −15 °C/10 nM gemcitabine combination condition.

When samples were treated with the combination of 100 nM gemcitabine pretreatment and freezing to −15 °C, a further decrease in cell viability was noted compared to either condition alone as well as the 50 nM combination samples. Specifically, PANC-1 cells exposed to 100 nM gemcitabine for 48 h followed by a single freeze at −15 °C resulted in a decrease in cell survival to 60.9% (±1.8), which was significant when compared to −15 °C freeze alone (93.9.0% (±1.4); *p* < 0.01(#)), 100 nM gemcitabine alone (97.8% (±1.4); *p* < 0.01), or the −15 °C/50 nM gemcitabine combination condition (72.3% (±2.7); *p* < 0.01). As with the 50 nM gemcitabine/−15 °C freeze combination, assessment of the PANC-1 survival over the 7-day recovery period revealed a continued decline in the −15 °C/100 nM gemcitabine combination sample survival of 5.6% (±0.3), which was significant when compared to the day 7 viability of −15 °C freeze alone (123.7% (±1.6); *p* < 0.01), 100 nM gemcitabine alone (25.7% (±1.3); *p* < 0.01(&)), or the −15 °C/50 nM gemcitabine combination condition (20.7% (±1.1); *p* < 0.01(@)). This represented a ~3.6-fold increase in cell death versus 100 nM gemcitabine alone and a 2.7-fold increase compared to the −15 °C/50 nM gemcitabine combination condition.

#### 3.3.2. Impact of Adjunctive Oxaliplatin Pretreatment and Mild Freezing

**Figure 5:**
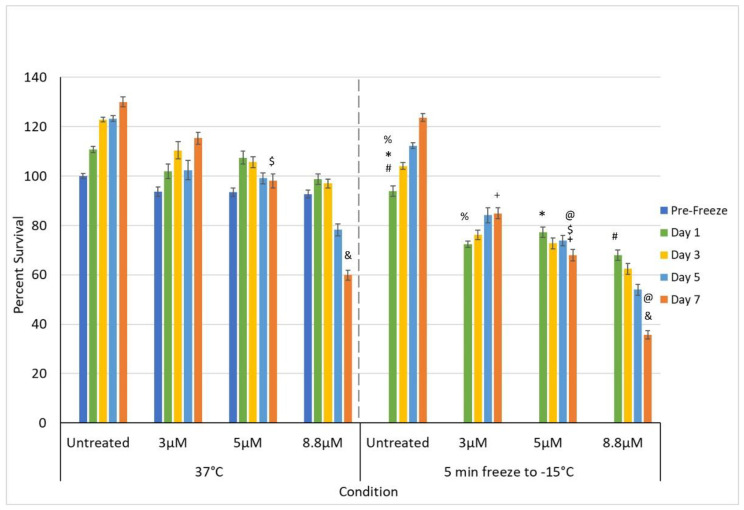
Effect of adjunctive oxaliplatin pretreatment in combination with freezing on PaCa cell survival. Samples were pre-treated with 3, 5, and 8.8 µM oxaliplatin for 48 h and drug removed prior to freezing to −15 °C. Sample viability was compared to freezing and oxaliplatin treatment alone. Data reveal a significant improvement in cell death following the combination of 8.8 µM oxaliplatin and −15 °C, whereas 3 and 5 µM oxaliplatin and −15 °C samples showed no to minimal benefit (@, #, $, %, &, *, + = *p* < 0.01).

In addition to gemcitabine pretreatment, investigation of the combination of low-dose oxaliplatin pretreatment and mild freezing was also conducted. The 3, 5, and 8.8 µM conditions were selected as, while none of them were completely lethal, they covered a range of clinical dose equivalents of ~1/3, 2/3, and clinical (e.g., 45, 73, and 130 mg/m^2^, respectively). Dose response studies revealed this range also yielded a varying level of impact from minimal cell death and full recovery (3 µM) to continual decline with moderate cell survival (8.8 µM = 59.9% (±2.0)) following 7 days of recovery (Figure 3b). The combination of 3 µM oxaliplatin and freezing yielded an initial decrease in PANC-1 survival compared to either treatment alone. However, the remaining population was able to recover over the 7-day assessment interval (Figure 5). Specifically, PANC-1 cells exposed to 3 µM oxaliplatin for 48 h followed by a single freeze at −15 °C resulted in a decrease in cell survival to 72.5% (±1.2), which was significant when compared to −15 °C freeze alone (93.9.0% (±1.4); *p* < 0.01(%)) or 3 µM oxaliplatin alone (93.7% (±1.8); *p* < 0.01). Continued assessment of PANC-1 survival over the 7-day recovery period revealed a slow regrowth of the surviving population reaching 84.9% (±2.2) of the pretreated controls. When samples were treated with the combination of 5 µM oxaliplatin pretreatment and freezing to −15 °C, a similar decrease in initial cell viability was noted at day 1 as with 3 µM oxaliplatin/−15 °C samples (77.3% (±2.1) vs. 75.5% (±1.2), respectively; *p*= 0.089). While not statistically different from the 3 µM oxaliplatin/−15 °C samples, this again was significant from either 5 µM oxaliplatin or −15 °C freezing alone (*p* < 0.01(*)). In contrast to the 3 µM oxaliplatin/−15 °C samples, assessment of sample viability following the 7-day recovery interval revealed a continued decline in overall viability to 68.0% (±2.4(+)) (*p* < 0.01) versus the recovery noted in the 3 µM oxaliplatin/−15 °C condition. 

When samples were treated with 8.8 µM oxaliplatin and then frozen to −15 °C, a similar decrease in day 1 cell viability was again observed as with the 3 and 5 µM oxaliplatin/−15 °C samples (68.0% (±2.1) vs. 75.5% (±1.2) and 77.3% (±2.1), respectively). As with the 5 µM oxaliplatin/−15 °C samples, analysis over the 7-day recovery interval revealed a continued decline in sample viability to 35.7% (±1.8) in the 8.8 µM oxaliplatin/−15 °C samples. This decline resulted in a significant decrease in overall survival compared to the 3 and 5 µM oxaliplatin/−15 °C conditions as well as the freeze and 8.8 µM oxaliplatin alone conditions. Specifically, overall viability at day 7 for the 8.8 µM oxaliplatin/−15 °C samples was 35.7% (±1.8) compared to 59.9% (±2.0) in 8.8 µM oxaliplatin alone (*p* < 0.01 (&)) and 68.0% (±2.4) for the 5 µM oxaliplatin/−15 °C samples (*p* < 0.01(@)). This represented a ~68% increase in cell death versus 8.8 µM oxaliplatin alone and a 90% increase compared to the 5 µM oxaliplatin/−15 °C combination condition.

### 3.4. Assessment of Caspase Activity following Treatment

**Figure 6:**
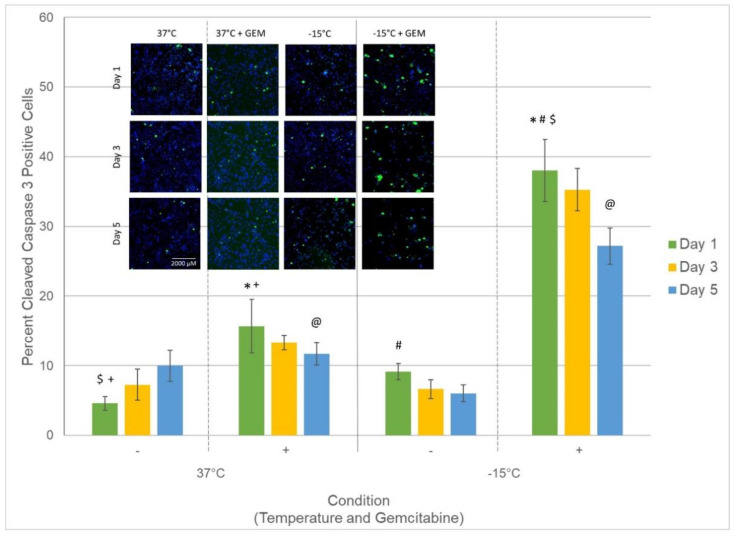
Analysis of percent of PaCa cells with active caspase-3 following treatment. Samples were treated with freezing to −15 °C, 50 nM gemcitabine, or the combination. The percent of cells with cleaved caspase-3 (active caspase-3) present within the nucleus was assessed at 1, 3, and 5 days post-treatment to determine the level of involvement and timing of apoptotic cell death associated with each treatment. Data reveal a significant increase in the number of cells with cleaved caspase-3 in gemcitabine/−15 °C combination samples compared to either treatment alone. The increased and prolonged nuclear presence of active caspase-3 indicates increased apoptotic activity in combination samples, which is believed to be responsible for the observed increased cell death. Insert: Representative mosaic fluorescent micrographs, acquired using the CX5 high throughput image analysis system, of the remaining cells (blue) and subpopulation of cells with active caspase-3 within the nucleus (green) (Scale Bar = 2000 µM). Data in bar graph represent the average (±SD) of the analysis of the mosaic images acquired from 9 replicate samples. (@, #, $, *, + = *p* < 0.01).

Based on the significant increase in PaCa cell death observed following the combination of mild freezing and low-dose gemcitabine compared to either freezing or gemcitabine alone (day 1: Gem/−15 °C: 72.3% (±2.7) vs. −15 °C: 93.0% (±1.4) vs. 50 nM gemcitabine: 97.6% (±1.7)) coupled with the continued decline over the 7-day assessment interval, studies were conducted to determine if the combination treatment may be activating an enhanced apoptotic response resulting in the increased PaCa cell death. To this end, we examined the level of active caspase-3 (cleaved caspase-3) within the nuclease of PaCa cells following freezing to −15 °C, 50 nM gemcitabine, or combination treatment (Figure 6). Analysis revealed a low basal level (<10%) of cleaved caspase-3 (active) in control samples over the initial 5-day recovery interval. Caspase-3 activity was found to increase in gemcitabine only samples compared to controls and was most noted 1-day post-treatment (15.6% (±3.8) vs. 4.5% (±1), respectively (*p* = 0.01(+)). Following freezing to −15° C alone, a slight increase in the number of cells with active cleaved caspase-3 within the nucleus was observed (9.3% (±1.1) vs. 4.5% (±1), respectively (= 0.03)); however, the number of cells with active caspase-3 was observed to decrease to controls by day 5. Interestingly, in PaCa samples treated with gemcitabine followed by freezing to −15 °C, a significant increase in the percent of cells with active caspase-3 within the nucleus compared to all other conditions was noted. Specifically, in the combination treated samples, 38.1% (±4.5)) of the remaining cell population was found to have caspase-3 activity within the nucleus. The percent of cells with nuclear caspase-3 activity was further observed to remain elevated compared to the other conditions throughout the 5-day recovery interval (day 3: 35.2% (±3) and day 5: 27.2% (±2.6)). This was significant compared to gemcitabine and −15 °C alone samples (*p* < 0.01 (*, #, @))

## 4. Discussion

There is little question that new approaches and technologies are needed for the treatment of PaCa. The standard treatment for PaCa patients is surgical resection or chemotherapy followed by chemo-radiation therapy, resulting in a median survival of six to twelve months [1,3]. Yet, in about 50% of patients with no metastases, tumor resection is not feasible because of vascular invasion, proximity to bile duct, poor general health, or lacking surgical access [37,38,87,88]. Therapeutic options for unresectable PaCa are limited to chemotherapy and chemo/radiotherapy. The growing annual incidence (>490,000 globally) and a 5-year survival rate of <10% (>460,000 deaths projected for 2021), which despite extensive research has not changed in over two decades, clearly argues that new approaches to treat PaCa are needed [1,3,89,90]. Ablation thermal therapy, including cryoablation and RFA, offer such potential. 

In this study, we investigated the survival of a PaCa cell line (PANC-1) following freezing in an effort to identify the minimal lethal temperature (dose) necessary for complete cell destruction. PANC-1 cells have been reported to be more resistant to freezing and heating injury as well as chemotherapeutic treatment than other PaCa cell lines (e.g., BxPC3 and MIA-PANC-2) [57,59,60,61,62,63], as such PANC-1 cells were utilized in an effort to identify a new potential treatment strategy for this challenging metastatic PaCa variant. Identification of the minimal lethal temperature could aid in the application of cryoablation to treat PaCa, enabling enhanced outcome and precision. Investigations into the impact of the combination of low-dose (sub-clinical) gemcitabine and oxaliplatin pre-treatment and mild freezing (−15 °C) were also conducted as studies have suggested the benefit of adjunctive drug/freezing in enhancing cancer kill (elevating the minimal lethal temperature) under conditions which when applied as a monotherapy (freeze or drug alone) are non-lethal [22,42,44,45,47,51,52,53,54,66,67,68,69,70,71,72].

Initial freeze response studies examined PANC-1 cell survival following exposure to a single freeze event applied using a cryoprobe in a TEM. Studies revealed exposure to ≥−15 °C resulted in minimal cell death whereas exposure to temperatures below −15 °C resulted in increasing cell death as the temperature decreased until <−25 °C was reached, wherein complete cell death was observed (Figure 1). These findings correlated well with previous reports wherein complete PaCa cell death has been reported to be attained in the −25 °C to −30 °C range (Figure 2). Given this, −15 °C was selected for subsequent combination therapy studies as increasing cell death following exposure to −15 °C would significantly increase the overall efficacy of a freeze procedure. Further, the −15 °C isotherm has been shown to fall within the hyperechoic rim visualized under ultrasound during a procedure. As such, the hyperechoic rim provides a visual reference point for determining where the lethal zone (−15 °C isotherm) is located in real time during a freeze procedure.

Studies continue to document the benefit of adjunctive treatment combining freezing with chemotherapy, nutraceuticals, or other agents in a number of cancers including prostate, breast, lung, and liver [42,44,45,47,51,52,53,54,66,67,68,69,70,71,72]. These and other studies have demonstrated that combinatorial approaches can increase the minimal lethal temperature necessary to kill cancer cells while offering the potential of reducing the overall negative side effects associated with traditional systemic chemotherapy. While the combination of cryoablation and low-dose chemotherapy remains in the investigational stages clinically, several published reports on the adjunctive approaches lend support to its potential [41,48,52,64,86]. Based on these reports and the current usage of gemcitabine and oxaliplatin to treat PaCa, we investigated the potential of combining a low-dose chemotherapy agent pretreatment with freezing. Pre-treatment with 50 or 100 nM gemcitabine (0.56 or 1.11 mg/m^2^) in combination with a single freezing event was found to increase the minimal lethal temperature to −15 °C, as illustrated by a significant increase in cell death on day 1 and complete cell death by day 7 (Figure 4). Similarly, pre-treatment with 8.8 or 10 µM oxaliplatin (clinical dose range) in combination with freezing to −15 °C resulted in a significant increase in cell death on day 1 post-freeze and near-complete cell death at day 7 (Figure 5). 

With the observed increase in cell death following combination treatment with −15 °C and 50 nM gemcitabine, studies were conducted to determine that the increased cell death was a result of increased apoptosis. To this end, samples were analyzed for the presence of active caspase-3 (cleaved caspase-3) following treatment. Our analysis focused on the assessment of cleaved caspase-3 within the nucleus of cells remaining following treatment. Assessment of nuclear cleaved caspase-3 was elected given caspase-3 is an executioner enzyme responsible for DNA cleavage in the terminal stage of apoptosis. Pro-caspase-3 (inactive form) is present in the cytoplasm and must be activated and transported into the nucleus as part of the apoptotic caspase cascade in order for caspase-mediated apoptosis to be completed. As such, assessment of cleaved caspase-3 within the nucleus is a key indicator of the involvement and manifestation of apoptotic cell death. Analysis of nuclear cleaved caspase-3 in samples following treatment revealed a mild increase in the percentage of cells with active caspase-3 in gemcitabine and −15 °C alone samples 1 day following treatment compared to controls (Figure 6). In comparison, the combination of 50 nM gemcitabine and −15 °C was found to result in a substantial increase in the number of cells with active caspase-3. Specifically, there was a ~150% increase in cells with nuclear caspase-3 activity compared to gemcitabine alone samples (*p* < 0.01(*)) 1 day following treatment and a 145% increase 5 days post treatment. This increase in caspase-3 activity was even greater when compared to freeze alone samples (D1 = 3.2-fold and D5 = 3.5-fold increase). The prolonged increase in active caspase-3 within the nucleus in combination samples is believed to be responsible for the observed increased cell death following combination treatment. Moreover, the observed prolonged increased caspase-3 activity is further believed to be responsible for the continued cell death observed in combination samples over the assessment interval.

Although the treatment of PANC-1 cells with gemcitabine or oxaliplatin at 37 °C resulted in a gradual decline in viability over the 7-day assessment period at each concentration evaluated, the combination of drug pretreatment and freezing significantly accelerated the rate of decline and, under select conditions (doses), complete cell death was achieved following the combination of low-dose agent pre-exposure and a single freeze at −15 °C (Figure 4). This is compared to the −25 °C necessary for complete cell destruction for freezing alone, whereas in the drug alone condition, complete cell death was not attained. When translating this to a clinical scenario, assuming an elliptical ice ball formed on a 3 cm long freeze zone cryoprobe, this increased the minimal lethal temperature to −15 °C, representing a ~70% increase in the ablative volume of an ice ball (from <40% within −25 °C to ~62% within the −15 °C isotherm boundary) based on the reported isothermal distribution within a typical argon-based JT cryolesion [27,91,92]. This increase in destruction could decrease the risk of cancer survival and recurrence in the periphery of a frozen mass, thereby increasing the likelihood of a curative outcome. Further, this could also have the benefit of expanding inclusion criteria to patients with tumors located in close proximity to vital structures, where the application of ultracold temperatures necessary for assured cancer destruction is challenging. For instance, one advantage of combination treatment may be to convert borderline and non-resectable patients to resectable. For example, typically resection is not attempted when there is arterial vasculature abutment or involvement. Cryoablation has been found not to freeze major vessels when located within the periphery of an ice ball where temperatures remain above −25 °C to −30 °C (nominally). It is important to note that the application of ultracold temperatures (<−30 °C, nominally) will result in vessel freezing. When a blood vessel is located within the periphery of a frozen mass, blood flow within the major vessel acts as a heat source and provides a natural protection mechanism. In these cases, as the icefront approaches a blood vessel, the “heat” prevents the vessel from freezing and creates a “divot” in the ice front wherein the vessel is not damaged as long as temperatures remain above −25 °C (nominally). As such, combination therapy, potentially cryoablation alone, may offer the ability to freeze up to an artery, destroy PDAC, and eliminate vasculature involvement, converting the tumor to resectable if desired.

In our study, the treatment of PANC-1 cells exposed to the combination of sub-clinical low-dose gemcitabine or oxaliplatin pre-treatment followed by freezing to −15 °C resulted in increased cell death. While both combinations showed promise, the gemcitabine/freeze combination resulted in a greater improvement in cell death when compared to oxaliplatin. Specifically, the combination of 8.8 µM oxaliplatin and freezing resulted in an accelerated decline in PANC-1 viability in combination samples to 35.7% viability by day 7 compared to 61.6% in drug alone samples and complete recovery in −15 °C only samples. In comparison, pretreatment with 50 nM gemcitabine in combination with freezing to −15 °C resulted in a significant increase in cell death on day 1 post-freeze compared to controls (*p* < 0.01 (*, +)) and a further decrease by day 7 to 20.7% (±1.1). Importantly, pretreatment with 100 nM gemcitabine in combination with freezing to −15 °C resulted in a significant increase in cell death on day 1 and day 7 compared to 50 nM (*p* < 0.01 (@)) with near-complete destruction (viability = 5.6% (±0.3)) by day 7 (Figure 4). The dose of 100 nM gemcitabine is equivalent to 1.11 mg/m^2^, or approximately 1/900^th^ of the 1000 mg/m^2^ single intravenous weekly clinical dose. Further, the results suggest that cryoablation may be beneficial when applied as a secondary treatment once a patient is undergoing standard-of-care chemotherapy treatment.

Gemcitabine’s primary mechanism of action is the inhibition of DNA synthesis [86], while oxaliplatin exerts its effects mainly through DNA damage [93]. Chemoresistance can occur within just weeks of treatment in PaCa [94,95], which is attributed in part to the accumulation of mutations in key genes that occur in the vast majority of PaCa. These include the oncogene K-RAS and the tumor suppressors CDKN2A, TP53, and SMAD4/DPC4 [96]. In a small study of PaCa patients with refractory disease treated with both oxaliplatin and gemcitabine, no significant effects were observed [97]. This highlights the importance of first-line treatments that address a multitude of cancer’s adaptive mechanisms [25]. Adjunctive therapies utilizing freezing show great potential as a multifaceted approach [25], especially given that cryotherapy is non-selective for cell cycle stage [98], in contrast to RFA. Another major benefit to cryoablation is that it has been shown to launch a secondary immune response attacking metastatic disease [10,25,28,99,100,101,102,103,104]. Baust et al. [22,23,25,66,105,106] and others [10,28,44,45,47,100,101,102,103,104,107,108,109,110,111,112,113] have detailed the benefit of combining cryoablation with various drugs and immunomodulators to increase efficacy and potentially target metastatic disease. 

While promising, this study is not without limitations. The primary limitation is that this study was conducted on an in vitro cell model using a single PaCa cell line (PANC-1). As such, these findings need to be further explored in vivo. Further, the in vitro nature of this study provides a near-optimal environment for cell recovery following treatment. In vivo, following freezing and thawing, additional stressors, including prolonged tissue ischemia, inflammatory response, and the activation of apoptosis, provide additional destructive events, thereby increasing the level of cell death. These factors may further increase the minimal lethal temperature. Another limitation is that the freeze interval was limited to a single 5-min freeze. Clinically, 10-min freeze procedures have been traditionally utilized for the treatment of other cancers. Studies have suggested that attainment of the minimal lethal temperature for 30 s to 1 min can achieve cell death and that longer holds do not yield increased cell death [27,79,80]. As such, shorter freeze intervals are possible if target temperatures are attained. As previously reported, the PSN-based cryoablation system utilized in this study is capable of delivering an ablative dose more quickly and more efficiently than typical JT-based argon and as such the 5-min freeze time was more appropriate [79]. Given this, combined with the push to improve outcome while reducing procedure times (increased efficiency), we elected to utilize the shorter freeze time. Lastly, challenges with extrapolating varying dosing estimates across models (direct application vs. systemic administration, etc.) also limit translation of these findings to a clinical setting. Despite these limitations, the results are encouraging and support the potential for exploring gemcitabine treatment with cryoablation in PaCa, especially as an alternative first-line modality in locally advanced disease. 

## 5. Conclusions

In conclusion, our findings suggest that the minimal lethal temperature for PaCa following cryoablation is between −20 °C and −25 °C. Combination studies demonstrated that pretreatment with sub-clinical doses of gemcitabine (<1/10) followed by freezing elevates the lethal temperature to −15 °C, wherein complete cell destruction can be obtained. Similar results were found when combining one-half clinical and clinical doses of oxaliplatin with freezing at −15 °C. The data suggest that this combinatorial approach yielded an elevation of the minimum lethal temperature for PaCa from −25 °C to the −15 °C range. Extrapolating these in vitro findings to an in vivo scenario, the data suggest that both freezing alone and in combination with gemcitabine or oxaliplatin has the potential to improve outcome while reducing comorbidities associated with freezing and/or chemotherapy and may provide a new strategy for the treatment of PaCa. While promising, these in vitro findings need to be examined in vivo prior to clinical utilization.

## Figures and Tables

**Table 1:** Analysis of ice ball size and lethality following freezing.

	Average Ice Ball Size		Average Zone of Lethality		
	Diameter (cm)	Volume (cm^3^)	Diameter (cm)	Volume (cm^3^)	% of Ice Ball
Day 1	2.56 (±0.12)	13.55	2.01 (±0.18)	7.11	52.4
Day 3	2.56 (±0.18)	13.05	1.19 (±0.18)	7.01	53.2

## Data Availability

Not Applicable.
